# Forgiveness: A note for psychiatrists

**DOI:** 10.4103/0019-5545.49459

**Published:** 2009

**Authors:** Prakash Gangdev

**Affiliations:** Department of Psychiatry, University of Western Ontario and Mood and Anxiety Disorders Program, Regional Mental Health Centre-London

**Keywords:** Awareness, forgiveness, mental health, psychiatrists

## Abstract

Although forgiveness has received a lot of attention in the past two decades and its role in physical and mental health is being increasingly recognized, psychiatrists are unaware of its therapeutic benefits. A literature review was conducted with a view to create awareness of the recent advances in forgiveness research. Although forgiveness has been shown to be beneficial, more research is required, especially in the psychiatric setting. The role of resentment and bitterness in the causation of psychiatric disorders remain largely unevaluated and requires further study.

“The man who opts for revenge should dig two graves.” (A Chinese Proverb)

Forgiveness is traditionally a concept that is embedded in religion and all the major religions discuss forgiveness.[1] Philosophers and ethicists have debated on this topic[2] and forgiveness has been conceptualized, both as a value and as a weakness.[3] The postconflict reconciliation phenomenon in primates indicates that human forgiveness has an evolutionary significance in that there is a need for adaptation by cooperation in order to maintain social stability, and this can only occur if revenge seeking is replaced by forgiveness.[4] Politicians who have been held in saint-like reverence, like Mohandas Gandhi, Martin Luther King, Jr., and Nelson Mandela all practiced forgiveness, and the Truth and Reconciliation Commission in South Africa is an example of state-mediated amnesty program driven by forgiveness. Equally telling are the genocides such as the one in Rwanda, where revenge instead of forgiveness was in operation. The role of forgiveness in peace has been reviewed by O'Connell.[5]

The aversion of the social sciences to forgiveness was eroded with the publication of a book (Forgive and Forget: Healing the Hurts We Don't Deserve) by Lewis Smede, a theologian, who spurred an interest that led to empirical studies. Developmental, social, health, and personality psychologists, all began studying and promoting forgiveness.[6] Clinical applications of forgiveness as a therapeutic intervention were also published.[7] The International Forgiveness Campaign and funded research on forgiveness in the past two decades have created a greater awareness of forgiveness.[8] The Internet offers numerous resources and a number of organizations are engaged in promoting forgiveness, both as a sociopolitical and as a clinical intervention.[9–11]

A further impetus has been given to forgiveness by the recent developments in the Positive Psychology movement, with forgiveness being recognized as a positive psychological attribute.[12] Likewise, growing interest in spirituality has also created an increased awareness of forgiveness.[13] Moreover, there is some recognition that forgiveness may be an important component of psychotherapies as well. Psychoanalysts,[14–16] dialectical behavior therapists[17] and cognitive behavior therapists[18–19] have all recognized the importance of forgiveness in healing. The role of personality functioning in forgiveness has also been investigated.[20]

## BENEFITS OF FORGIVING

Forgiveness is associated with improved physical health[21–24] and mental health.[25–29] Psychophysiological[23] and neuroimaging[30] studies demonstrate the possible biological underpinnings of forgiveness. Forgiveness has been employed as an educational tool with beneficial effects[31–32] and has also been shown to be beneficial for victims of abuse[33–34] and unfaithfulness.[35] Thus, forgiveness is not only a virtue and a moral act, but it also has therapeutic potential. Admittedly, more research needs to be conducted in patients in a psychiatric setting.

## LACK OF AWARENESS AND SKEPTICISM

Not withstanding all the above, suggestions about the value of forgiveness is likely to be met with skepticism from both clinicians and patients. A few vignettes (with details deliberately altered) will illustrate the point:

- A patient had chronic depression which began after a family reunion where she met a cousin who had abused her many years ago. She had received antidepressants for several years without success, but after exploration, she decided to forgive her cousin. This was followed by a resolution of her depressed mood.
- On the other hand, patient F presented with a seven-year history of depression triggered by divorce from an abusive and unfaithful partner. Exploration revealed that she constantly ruminated and had vengeful thoughts, but felt guilty about them. She was unable to let go and a suggestion to consider letting go was not accepted. She expressed disbelief that a psychiatrist could make such a suggestion.
- Patient M had chronic, unremitting depression despite treatment for years with antidepressants, cognitive behavior therapy, and ECT. He could not forgive his long-dead grandfather for physical and emotional abuse when he was a child, and constantly ruminated and felt resentful and bitter. He also declined the suggestion to forgive his dead grandfather and let go of his anger as he did not believe that it would be beneficial.
- Patient F2 reported a depressed mood accompanied by extreme anger and thoughts of violence towards her colleagues who mistreated her. She was initially agreeable to the suggestion of forgiving her colleagues but later changed her mind.
- Another patient had unremitting depression and anger triggered by work problems and was unwilling to consider forgiveness as her case worker did not agree.

Surprise and skepticism were expressed by other health professionals at case conferences for these patients, and at a journal club, when a paper on forgiveness was presented to a group of psychiatrists there was a similar lack of awareness. Likewise, in a discussion with therapists working in a trauma program, a large number were very opposed to the idea of forgiveness as being of any therapeutic value for their patients.

Skepticism and lack of awareness appears to be multifactorial. Some, if not most psychiatrists do not read widely, and may not have access to theology, counseling, psychology, nursing, social work, and other related discipline-specific literature. Most of the empirical studies have been conducted by psychologists and professionals of other disciplines, and are not published in psychiatry journals. Literature on forgiveness in psychiatry is very sparse and has apparently not evoked any clinical, theoretical, or intellectual interest, as judged from their citations or published responses/comments. None of the surveyed treatment recommendation guides[36–38] or text books[39–42] make any reference to forgiveness as an intervention. Furthermore, the focus on medications and popular well-researched psychotherapies (probably due to the involvement of psychiatrists in the development/research of these psychotherapies), and some aversion to or avoidance of religion/spirituality (forgiveness being a part), coupled with a failure to acknowledge the role of anger/bitterness/resentment in the causation and perpetuation of mental health problems underlie the neglect of forgiveness by psychiatry. Also, all these problems are confounded by the lack of an adequate diagnostic framework that account for the role of resentment, a tendency to use personality disorder as an explanation for ‘treatment-resistant’ problems, and the use of DSM in research/practice[43] that disclaims any suggestions about etiology. There are however noteworthy exceptions which take cognizance of the importance of anger[44] and forgiveness.[45] Another reason may be that published evidence may not have been considered robust enough to be included in the ‘evidenced-based’ treatment guidelines. Moreover, reluctance in applying the findings of the intervention studies may also have to do with the fact that most of the studies were done on nonclinical populations. Finally, it may have to do with the expectation of the ‘patients’ that physicians will offer or recommend medical rather than religion/spirituality-based explanations/interventions for their problems.

## CLINICAL IMPLICATIONS FOR PSYCHIATRISTS

Given the neglect and a lack of awareness, it is time to call the attention of psychiatrists to forgiveness. Notwithstanding the lack of consensus about the definition of forgiveness and the associated theoretical models, it may be defined as releasing or foregoing of bitterness and vengeance by a victim toward the perpetrator of an offence, while acknowledging the seriousness of the wrong.[46] Forgiveness is distinct from pardon, condoning, forgetting, and reconciliation.[7] Self-forgiveness is the willingness to abandon self-resentment in the face of one's own acknowledged objective wrong, while fostering compassion, generosity, and love toward oneself.[47] A slight departure (with possible significant implications for psychiatry) occurred in the definition provided by DeShea and Wahkinney: self-forgiveness is a process of releasing resentment toward oneself for a *perceived* transgression and wrongdoing.[48]

A range of emotions (anxiety, hurt, sadness, hostility, and anger), cognitions (revenge seeking, ruminations, and cognitive rehearsal), and behaviors (grudge-bearing, avoidance of the perpetrator, demands for atonement) occur when a person is mistreated or victimized. Depending on the context and the personality factors, there may be open expression of the feelings, or the anger is muted, paving way for resentment[49] which has been linked to psychopathology and may underlie various psychiatric conditions.[7,50] Resentment *per se* is not considered a diagnosis.[51] Recently, Linden[52] suggested that bitterness could lead to a distinct adjustment disorder which he named, “Posttraumatic-Embitterment Disorder”, and compared it with other mental disorders.[53]

## THE INDIAN PERSPECTIVE

All religions practiced in India emphasize the value of forgiveness. Religion plays a significant part in our lives and seeking forgiveness and forgiving are easily understood concepts to most Indians. The Mahabharata glorifies forgiveness.[54] Jains observe Kshamavani Divas seeking and granting forgiveness.[55] Forgiveness is also mentioned in Buddhist, Christian, and Sikh scriptures, and Quran.[56] The stories of a Buddhist monk who upon being chopped, only experienced compassion for the king who ordered his torture, and King Ashoka extolling forgiveness after his conversion to Buddhism are well-known examples in Buddhist literature.[57]

While it is a practice in India to seek forgiveness (“*maaf kar do*” *in Hindi*”—whether arising from genuine remorse or just an over-learned response), *e.g*., if you do not wish to or are unable to give alms to a mendicant or if you accidentally touch the body of a person with your feet, in contrast, throwing acid on women by men who are rejected by them, religious riots, and gang wars are familiar examples of extreme anger/unforgiving attitude, often depicted in numerous Bollywood movies. Interestingly, as depicted in ‘Gandhi, My Father,’ it appears that Gandhi*ji* was unable to forgive his son, Ramdas, for his transgressions and conversely, Ramdas did not forgive Gandhi*ji* for ‘neglecting’ him, resulting in tragic personal consequences for both. On the other hand, the recent meeting between Priyanka Gandhi and the woman convicted for conspiring to assassinate her father, the late Prime Minister Rajeev Gandhi, is an important, recent example of forgiveness. Interestingly, in a study of Indian women with HIV/AIDS, the participants blamed their parents for marrying them to an inappropriate person, rather than their spouse for the infection, and forgiving their parents was of special significance.[58] It is surprising, therefore, that forgiveness has not received much attention in clinical practice or research in India. Over-reliance on international classification systems and research, coupled with a predominant emphasis on biological factors and medications may underlie the neglect of the value of forgiveness.

A new opportunity awaits psychiatrists who work with people who have been wronged and whose resentment may be both disabling but still understandable and justified. On the other hand, the possibility of addressing *perceived* wrongs (by self as in depression, and by others as in a range of psychiatric disorders, including psychotic disorders) also exists although this is at variance with the true intent and purpose of forgiveness, as defined by social scientists/philosophers. Regardless of the ‘diagnosis’ or the diagnostic status of resentment and vengeance, they need to be identified and addressed. Worthington[59] urges us to share his passion for forgiveness, and reminds us to look for the proof in our own life. However, caution is urged in the use of forgiveness[3,13,60] and the patient's personal and cultural beliefs about forgiveness and the nature of the offence are of prime consideration.[46,61] Moreover, research is limited by the fact that there is yet no consensus on the definition and measurement of forgiveness, which has led to several definitions and scales to measure forgiveness. The various forgiveness measures include self-reports by participants as to how they might react to a hypothetical transgression or their views on forgiveness. It is difficult to predict how they will react in a real life transgression based on their response to hypothetical scenarios, and if the actual transgression is studied, again it is possible that, depending on the nature of the next transgression, they may or may not have the same response.

A number of additional barriers may further limit the implementation of forgiveness in clinical setting. Forgiveness may be misunderstood as a weakness and may lead to the notion that one's right to seek justice is waived. It may be deemed as conditional only to a sincere apology and if the offender is absent, it may be thought that the question of forgiveness does not exist. On the other hand, it may encourage the offender to re-offend. Moreover, (sweet) revenge may be thought of as a more fitting response than forgiving.

In summary, it is time that psychiatrists familiarize themselves with the benefits of forgiveness as a therapeutic intervention and also, with its limitations. The use of forgiveness as an intervention in psychiatric disorders will be facilitated by: a) research into the connection between resentment/bitterness/anger in the wake of transgression and psychiatric disorders, and b) intervention studies in psychiatric disorders. Cultural norms and beliefs will need to be taken into account in any future research.
